# Dynamic evaluation of outdoor thermal comfort in an urban park situated in a tropical region

**DOI:** 10.1007/s00484-026-03359-2

**Published:** 2026-10-30

**Authors:** L. C. Lima, S. M. Leder, E. L. Krüger

**Affiliations:** 1 Department of Architecture and Urban Planning, Federal University of Paraiba, João Pessoa, Brazil; 2 Department of Civil Construction, Federal University of Technology of Parana, Curitiba, Brazil

**Keywords:** Dynamic thermal comfort, Climate walk, Thermal indices

## Abstract

This study employed a longitudinal design along a 1,390-meter walking route in an urban park in a hot, humid climate. Environmental variables were continuously monitored using a backpack-mounted device equipped with low-cost microclimatic sensors. Subjective thermal environment assessments were conducted at six designated points along the route, with questionnaires administered to 103 participants across wet and dry periods. Comparison of subjective assessments with the Universal Thermal Climate Index (UTCI) revealed discrepancies in perceived thermal sensation. Rather than an inherent metric inaccuracy, these findings highlight the index’s predictive limits and scale-resolution challenges under dynamic field conditions. While subjective assessments showed statistically significant correlations with air temperature, relative humidity, and the UTCI, the practical magnitude of these relationships was weak due to the repeated-measures design. This indicates that direct microclimatic variables alone explain limited variance in transient human comfort. Point-by-point comparative analysis revealed that spatial factors profoundly dictate thermal perception. Notably, densely shaded locations produced a sultry canopy effect during overcast, wet conditions, in which restricted airflow and trapped humidity elevated thermal stress despite lower ambient air temperatures. Ultimately, the study underscores the importance of capturing sequential thermal experiences, environmental contrast, and cumulative metabolic demands, particularly during the final uphill spatial transition. This research advances the evaluation of outdoor thermal comfort in the Global South through affordable dynamic monitoring and clarifies the complex microclimatic role of urban parks.

**Supplementary Information:**

The online version contains supplementary material available at https://doi.org/10.1007/s00484-026-03359-2.

## Introduction

Research on thermal comfort in outdoor spaces has intensified in recent years and is projected to continue to grow due to the challenges posed by climate change. Estimating thermal conditions in urban environments remains complex as microclimatic variables in outdoor settings are less controllable than indoors (Gobo and Galvani 2016). Outdoor thermal conditions significantly influence quality of life, particularly in large cities where public urban spaces shape daily activities. To evaluate the outdoor thermal environment, researchers frequently employ thermal indices (Fanger 1970; Höppe 1999), which serve as vital tools for quantifying environmental quality and minimizing risks associated with heat or cold stress. Among these, the Physiologically Equivalent Temperature (PET) (Höppe 1999) and the Universal Thermal Climate Index (UTCI) (Fiala et al. 2012) are widely utilized. Specifically, the UTCI functions as an advanced thermophysiological reference tool based on a multi-node human heat balance model, designed to assess heat stress and physiological strain under given meteorological reference conditions (Fiala et al. 2012; Bröde et al. 2012). However, as an index of thermal stress, the UTCI primarily reflects standardized whole-body physiological strain rather than functioning as a direct psychological measure of subjective thermal comfort, thermal pleasure, or transient perception.

In addition to selecting thermal comfort indices, the research protocol and methodology significantly influence research outcomes. Field studies typically employ either static approaches, which rely on point measurements and cross-sectional analyses, or dynamic approaches, such as mobile monitoring with wearable sensors that capture changes in environmental conditions and human responses over time (Aghamolaei et al. 2023; Liu et al. 2023). This methodological distinction is important because it shapes the depth of understanding of thermal adaptation processes and the variability of thermal sensations in outdoor environments (Ng and Cheng 2012; Wang et al. 2018). Furthermore, dynamic monitoring of specific areas enables higher-granularity datasets, providing greater resolution than fixed-point data collected at the pedestrian level (Cureau et al. 2024). This approach has led to increased interest among researchers in wearable environmental monitoring systems (Chokhachian et al. 2018; Nouman et al. 2019; Cureau et al. 2022).

However, the high cost of commercial gear limits the expansion of research on dynamic monitoring of microclimatic variables with wearables. A promising alternative involves low-cost sensors integrated with microprocessors (Kimmling and Hoffmann 2019; Romero et al. 2020; Ali et al. 2016; Callejas et al. 2024). According to Romero (2020), the low-cost approach prioritizes ease of use, customization, assembly, and replicability. Portable and affordable stations offer significant advantages over fixed stations by integrating multiple sensors for microclimatic and environmental monitoring within a single device. Backpack-style stations can be versatile and pedestrian-friendly in various urban contexts (Callejas et al. 2024; Ihlenfeld 2024; Lima 2024). In contrast, dynamic monitoring using vehicles, bicycles, or trolleys may face challenges in accessing certain pedestrian areas within the urban network.

Low-cost microcontroller-based devices also use open-source software and programming languages designed for prototyping platforms such as Arduino and Raspberry Pi, which rely on accessible source code and adaptable development frameworks (Nouman et al. 2019). The widespread availability of affordable sensors supports research initiatives in developing countries and projects with limited funding by favoring replicability, while also enabling greater customization and simplifying component replacement, which often have shorter operational lifespans (Krüger et al. 2023).

Most outdoor studies continue to employ cross-sectional methods, with single-vote questionnaires administered to large populations without repeated measures over time while moving. In contrast, longitudinal studies evaluating individuals over time under continuous environmental exposures remain scarce. Dynamic thermal comfort research emphasizes the temporal evolution of human-environment interactions, explicitly accounting for changing microclimatic conditions alongside occupants’ physiological and perceptual responses. As demonstrated by recent methodological frameworks, “climate walks” are essential to transition from static observations to capturing a pedestrian’s continuous thermal journey through space and time (Lau et al. 2026; Vasilikou and Nikolopoulou 2020). Emerging evidence indicates that spatial movement, transient thermal exposures, short-term thermal history, and cumulative metabolic heat substantially influence subjective thermal sensation and comfort perception (Lau et al. 2019; Dzyuban et al. 2022).

Consequently, the primary purpose of this study is to shift the analytical focus from isolated, point-based thermal assessments toward a comprehensive evaluation of the sequential thermal experience. By employing continuous walking and point-to-point comparisons, this research aims to capture participants’ transient thermal experiences during spatial transitions by integrating environmental contrast, thermal history, psychological expectation, and accumulated metabolic heat. In this context, a longitudinal dynamic thermal comfort experiment was conducted, combining in situ microclimatic measurements with mobile monitoring equipment during a climate walk over rough terrain. Microclimatic and thermal stress reference data (UTCI) were collected using a portable low-cost station, while subjective right-here, right-now surveys were administered to pre-selected participants.

The study emphasizes the relevance of urban parks with forest remnants from reforestation as thermal respite areas in cities of the Global South. It contributes to the body of knowledge on outdoor thermal comfort research using affordable monitoring equipment in tropical regions, highlighting how the dynamic character of climate walks and transient exposure analyses provide a more accurate reflection of human thermal adaptation in real-world urban transit.

## Method

The experiment was conducted in an urban park within an Atlantic Forest fragment in a city with a hot, humid tropical climate in northeastern Brazil. The main methodological components are the characterization of the study area and measurement transect; microclimatic monitoring procedures; subjective thermal perception assessment instrument; and data processing and analysis procedures.

### Characterization of the study area and measurement transect

The study site was a botanical zoo park covering 26.8 hectares in the city of João Pessoa, Brazil (7°06’51.32’’ S, 34°52’38.70’’ W). Located within the Atlantic Forest biome, the park hosts a variety of endemic and exotic fauna and flora and is frequented by residents and tourists from different regions for nature appreciation, physical activities, leisure, recreation, social interaction, and rest. João Pessoa has a Tropical savanna (As) climate under the Köppen-Geiger classification (Kottek et al. 2006), characterized by a narrow year-round temperature range, with an annual mean temperature between 24 °C and 26 °C, maximum air temperatures ranging from 28.7 °C to 31.2 °C, minimum air temperatures from 21.9 °C to 25.1 °C, average relative humidity of approximately 80%, and annual precipitation of about 1800 mm. Seasons are primarily defined by precipitation: the wet season is characterized by increased cloud cover, whereas the dry season is characterized by clear to partly cloudy skies (Alvares et al. 2014; INMET 2021).

The Park features varied topography, with tree vegetation covering approximately 75% of the area. Land use encompasses grassy areas, water bodies, pathways, and several built structures. The selection of points along the route was based on criteria including travel distance, accessibility, estimated time to complete the route, participant safety, pavement type, terrain, vegetation density, exposure to solar radiation, and the presence of water bodies and buildings.

**Fig. 1:**
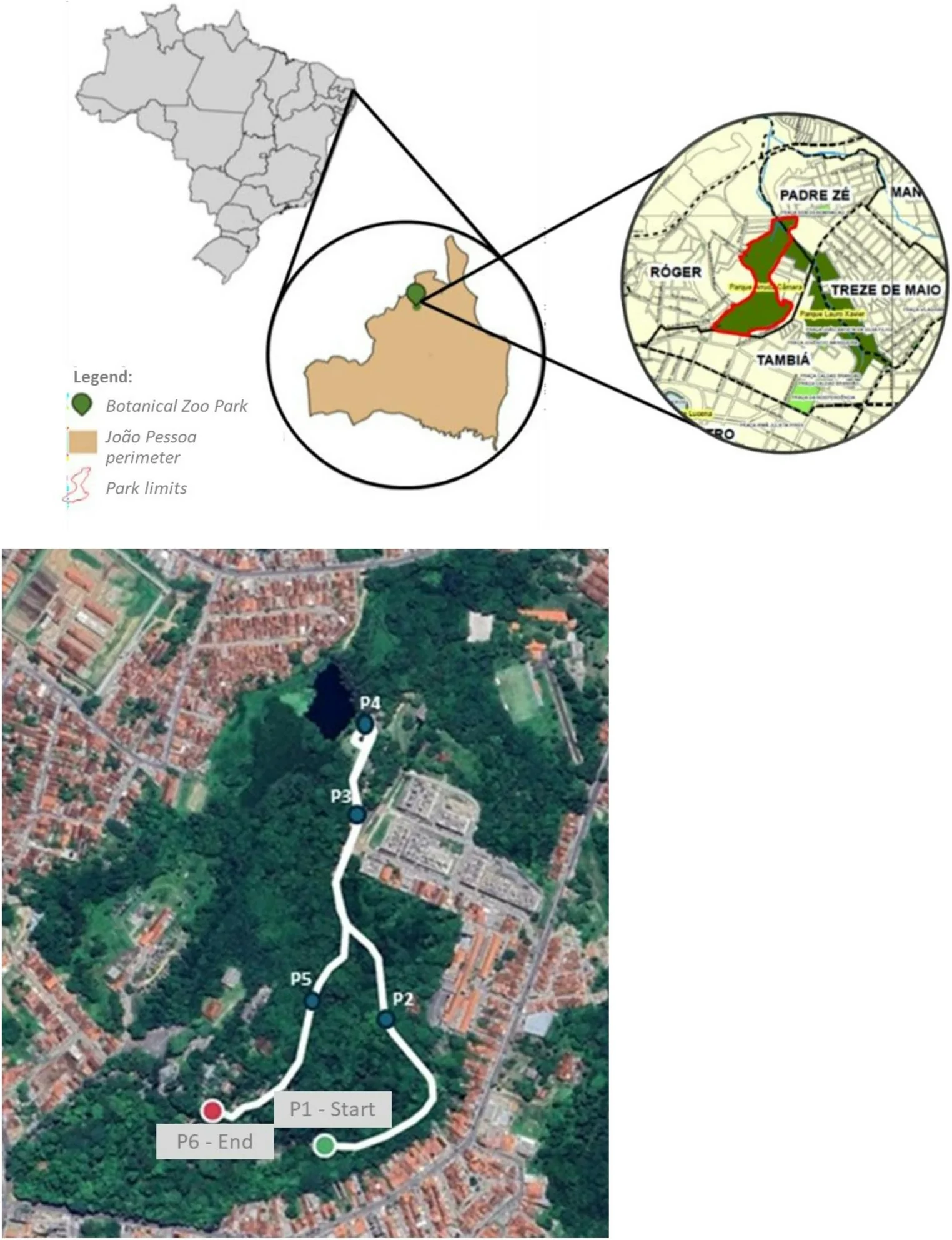
– Location map and point locations within the Botanical Zoo Park

The measurement route includes six stopping points (P1-P6; Fig. 1), totaling 1,390 m and featuring a positive elevation gain of 13 m. Point 1 is situated at the beginning of the park trail, where the walkway is paved with impermeable cobblestones, with a Sky View Factor (SVF) of 0.403 and robust vegetation, including shrubs and trees between 10 and 20 m in height, that provide extensive shade. Point 2 differs from Point 1 not only in its pavement material, consisting of permeable, exposed compacted sandy soil, but also markedly by exhibiting a considerably lower SVF of 0.174, highlighting distinct conditions regarding sky exposure, canopy shading, and radiation environments between the two locations. Point 3 is located near a park gathering area, adjacent to snack bars, the boarding and alighting area for the tractor-pulled train, and a large retaining wall at the park boundary. At this location, the walkway is impermeable and constructed of interlocking concrete paving. The vegetation is sparse, composed of shrubs and trees, resulting in alternating sunlit and shaded areas. Point 4 is positioned near a pond, with an impermeable surface covered by interlocking concrete paving. This is the most open area of the park, characterized by grassy spaces and fewer trees, which results in the highest exposure to solar radiation. Point 5 is located along a trail in the park’s lowest area, which is identified as a wetland. The trail alternates between sections of compacted soil, stones, grass, and narrow wooden walkways that cross small watercourses. The vegetation is dense, with several medium- and large-sized trees, making this the most shaded point along the route. Point 6, the final point along the route, is situated near a recreation area. The walkway is impermeable and paved with granite cobblestones. The tree vegetation is tall but not dense, resulting in both shaded and sunlit areas.

Photographic documentation of the points was conducted using a conventional smartphone camera with 4 K resolution. Sky View Factor (SVF) images were obtained with a 4 mm fisheye lens attached to a full-frame Nikon D90 camera, mounted on a tripod at 1.20 m above ground level, facing the sky, and with the camera top oriented toward true north. Shadow masks and the SVF index were generated using Gap Light Analyzer, an open-source software commonly used in vegetation image analysis to evaluate light distribution parameters (Table 1).

**Table 1:**
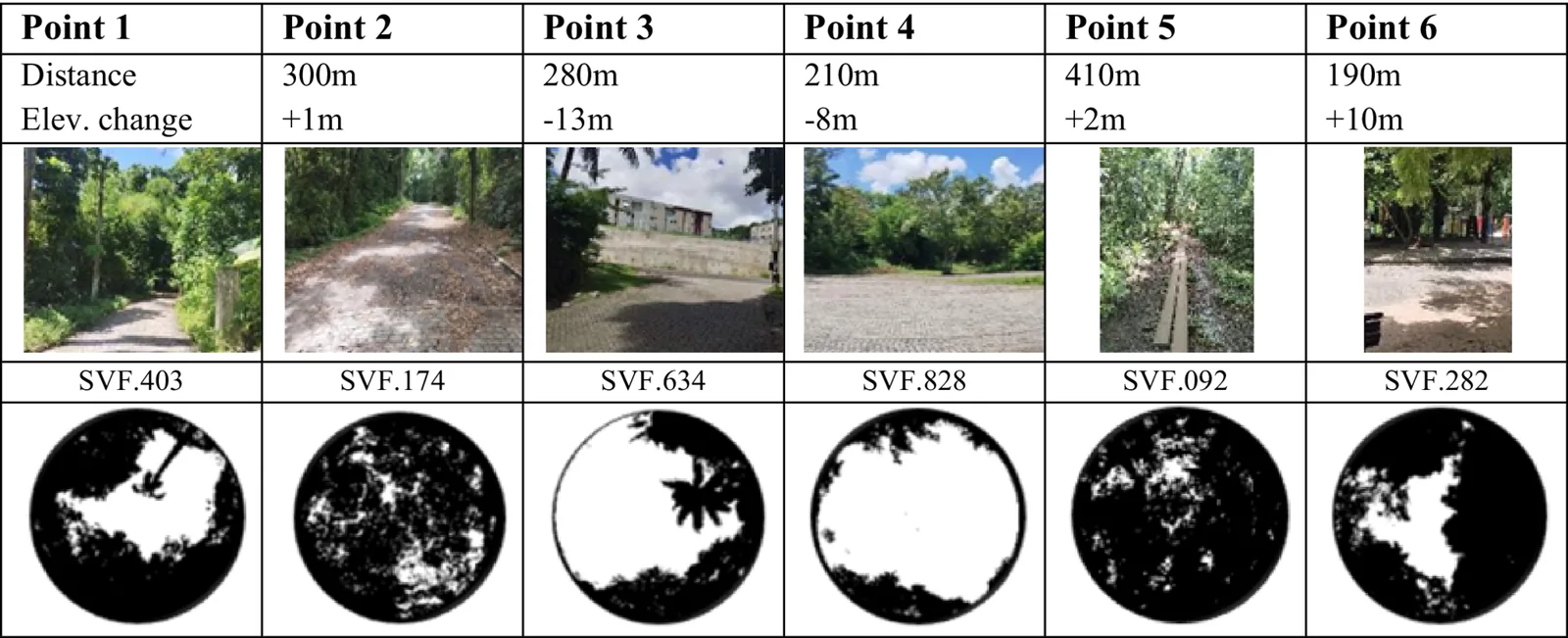
Points’ features

### Microclimatic monitoring

**Fig. 2:**
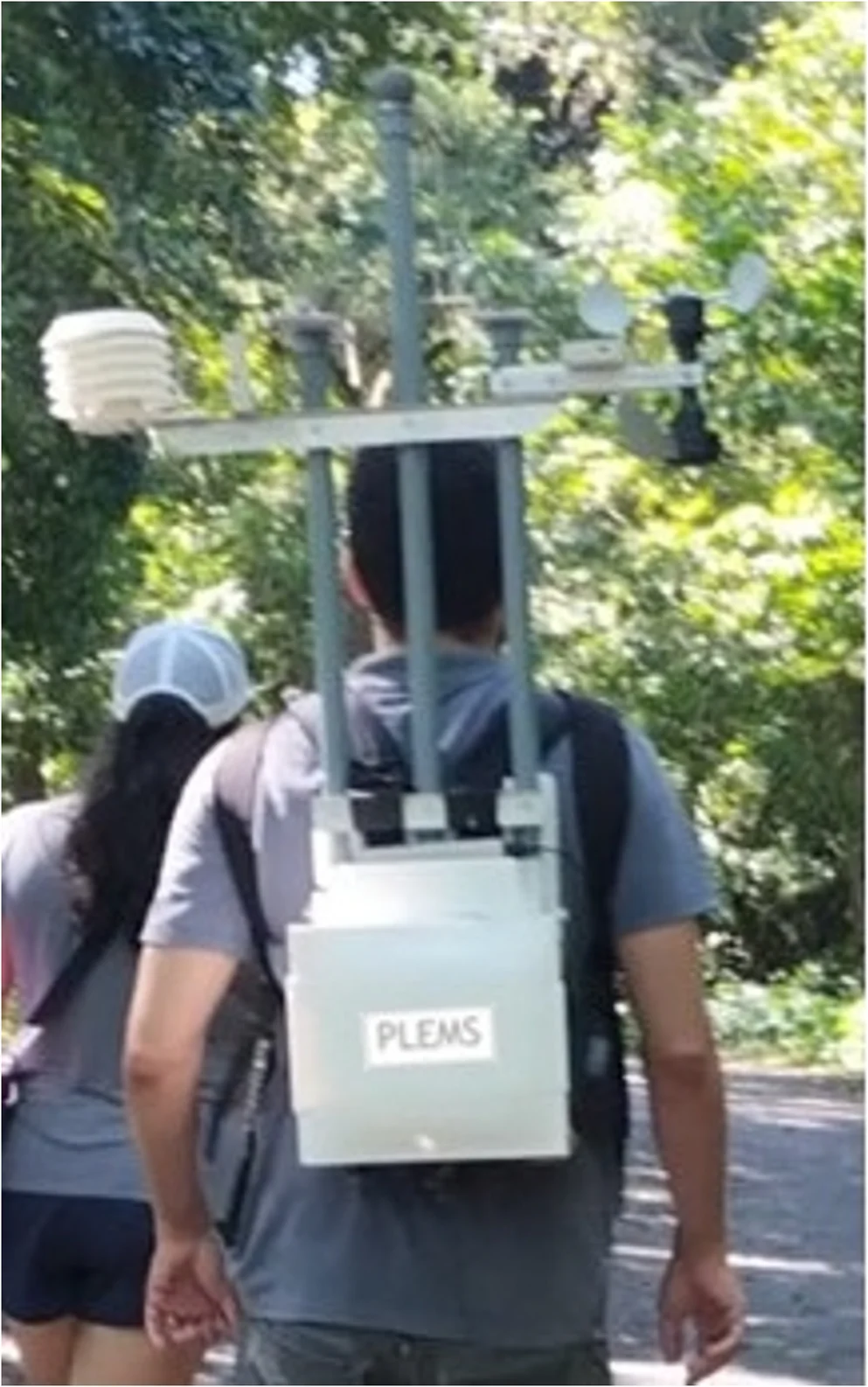
– PLEMS at the study site

The primary equipment used in this study was the Portable Environmental Monitoring System (PLEMS), a monitoring backpack developed collaboratively by researchers from the Federal University of Paraíba (UFPB) and the Federal Technological University of Paraná (UTFPR) for microclimatic monitoring in urban environments (Fig. 2). This device incorporates microclimatic sensors that measure air temperature, relative humidity, air velocity, and globe temperature (Table 2), all of which comply with the requirements for class C (comfort) specified by ISO 7726 (1998). The globe thermometer integrated into the PLEMS was fabricated from an official 40 mm table tennis ball, originally white and subsequently painted light gray, following the guidelines outlined by Thorsson et al. (2007). Further information regarding the development, construction, and additional sensors of the PLEMS can be found in Ihlenfeld (2024), Callejas et al. (2024), and Lima (2024).

**Table 2:** – PLEMS’ sensor specifications

Sensor	Resolution	Measurement range	Accuracy	
Air temperature (°C)	0.01 °C	-40 to 85 °C		± 0.3 °C
Relative humidity (%)	0.02%	0% to 100%		± 2%
Air velocity (m/s)	0.02 m/s	0.2 to 37 m/s		± 0.5 m/s
Globe temperature (°C)	0.06 °C	-55 to 125 °C		± 0.5 °C

Air temperature (Ta), relative humidity (RH), air velocity (v), and globe temperature (Tg) were recorded dynamically along the transect. Microclimatic monitoring occurred during two distinct periods: the wet season (July and August 2023) and the dry season (December 2023 and January 2024). Monitoring was conducted for 11 days during the wet period and 9 days during the dry period. In both periods, data collection was limited to days with standardized atmospheric conditions, including clear skies, no precipitation, and low air velocity, between 9 am and 4 m.

During the wet period, PLEMS recorded data approximately every 1.5 min. After programming improvements, data were recorded every 1 min during the dry period. At each of the six measurement points, the PLEMS operator remained stationary, facing true north, for 5 min. Consequently, four records per point were collected during the wet period at approximately 1.5-minute intervals, and five records per point were collected during the dry period at 1-minute intervals.

Background meteorological data were collected from two independent weather stations. Official data were retrieved from the INMET A320 climate monitoring station on Campus I of the Federal University of Paraíba (at 7° 08’ 37” S, 34° 50’ 59” W; altitude 43 m). Local baseline data were recorded at starting Point 1 using a Davis Vantage Pro weather station.

### Subjective thermal perception

Alongside microclimatic monitoring, a subjective thermal perception survey was administered to preselected participants. The questionnaire comprised three modules: (1) personal and biometric data, including biological sex, height, weight, clothing, comorbidities, occupation, and residency in João Pessoa; (2) subjective perception of the current point, encompassing thermal sensation, comfort, and preference; and (3) subjective and comparative perception of the current point relative to the previous point. According to ISO 10,551 (2019), thermal sensation was measured on a 7-point scale ranging from − 3 (cold) to + 3 (hot), with 0 indicating neutral or comfort. Thermal comfort perception was assessed on a 5-point scale from 0 (comfortable) to 4 (extremely uncomfortable). Thermal preference was evaluated on a 7-point scale from − 3 (much cooler) to + 3 (much warmer), with 0 representing ‘neither warmer nor cooler’. The third module, which addresses subjective perception in relation to the previous point, reflects the longitudinal study’s dynamic nature. Subjective comparisons between consecutive points along the route were conducted to evaluate thermal perception at the current point relative to the previous point, using a 3-point scale: colder, similar, or warmer.

To enhance data storage efficiency and reduce response time, the questionnaire was digitized using Google Forms and accessed via smartphones. Participants were allotted five minutes at each point to complete the questions, minimizing the likelihood of errors. At the initial point, volunteers received the questionnaire links through a mobile application, provided informed consent by signing the Informed Consent Form (ICF), and completed modules 1 and 2. This initial phase lasted approximately 15 min, allowing for equipment stabilization and participant acclimatization. From the second point onward, participants completed modules 2 and 3 of the questionnaire. The questionnaires received approval from the Research Ethics Committee of the Health Sciences Center at the university (UFPB) (Process number 34913620.8.0000.5188, with approval certificate 6.018.641).

### Data processing and analysis procedures

Microclimatic variables and questionnaire data were digitized, organized, and statistically analyzed. Descriptive statistics for subjective variables (questionnaires), microclimatic variables (Ta, RH, v, Tg, and Tmrt), and the Universal Thermal Climate Index (UTCI) were presented as box plots.

The UTCI was calculated with Rayman Pro 3.1 software (Matzarakis et al. 2010). Wind speed measurements were obtained at a height of 1.90 m. As the UTCI calculation requires wind speed values at 10 m, the logarithmic conversion formula proposed by Bröde et al. (2012) was applied.

The mean radiant temperature was derived from global temperature values using the forced-convection equation recommended by ISO 7726 (1998).

Body mass index (BMI) was calculated for each participant based on measured weight and height. The metabolic rate was set to 165 W/m², corresponding to walking at 4 km/h, as specified in ISO 8896 (2004). Following the calculation of comfort indices and descriptive analyses of the three variable sets (subjective, microclimatic, and thermal comfort indices), the normality of the variables was evaluated using the Shapiro-Wilk test at a 95% significance level. Since neither the qualitative nor quantitative variables followed a normal distribution, Spearman’s correlation at the 95% confidence level was applied to assess associations among these variable groups.

## Results

Results are organized as follows: a general description of the sample; monitoring of climatic variables; assessment of thermal comfort using the Universal Thermal Climate Index (UTCI); subjective evaluation; and statistical correlations among subjective feedback, microclimatic variables, and UTCI results.

### General description of the sample

Table 3 summarizes the descriptive statistics for the sample. During the rainy season, 50 volunteers participated, while 53 participated during the dry season. Among the 103 volunteers, most were young adults. Of these, 41 were male, and 62 were female. The average weight was 74 kg (SD ± 18 kg), and the average height was 1.67 m (SD ± 0.07 m). In total, 10 participants reported comorbidities. Of these, 4 had obesity, specifically hypertension (4) and a herniated disc (2). Table 3 also reports the calculated body mass index (BMI), clothing insulation as noted during the campaign, and place of origin. Although not all participants were locals, only 7 came from cities with different climates. 

**Table 3:** – Sample characteristics

Parameter	Category	Wet season	Dry season	Total
Sex	male	19	22	41
	female	31	31	62
Age	20–29 y-o	21	21	42
	30–39 y-o	25	24	49
	40–50 y-o	3	5	8
	50–60 y-o	1	2	3
	> 60 y-o	0	1	1
Weight (kg)	avg	72	75	74
	min	47	49	47
	max	115	125	125
	SD	17	19	18
Height (m)	avg	1.66	1.67	1.67
	min	1.50	1.50	1.50
	max	1.86	1.83	1.86
	SD	0.07	0.07	0.07
Clothing insulation (Clo)	avg	0.44	0.35	0.39
	min	0.18	0.19	0.18
	max	0.62	0.61	0.62
	SD	0.13	0.13	0.13
BMI	underweight	1	2	3
	normal	28	24	52
	overweight	8	17	25
	obese	13	10	23

### Monitoring of microclimatic variables and UTCI assessment

#### Continuous baseline monitoring

Data collected from the A320 climate monitoring station of the INMET network, situated in João Pessoa approximately 2 km from the Botanical Zoo Park, are presented in Supplementary Material A. During the wet season, the mean daily global solar irradiance was 18,209 kJ/m², with ambient temperatures ranging from 23 °C to 29 °C and a mean relative humidity of 69% during monitoring hours (between 9:00 am and 4:00 pm). In the dry season, daily global solar irradiance increased to 23,641 kJ/m², daytime temperatures ranged from 26 °C to 31 °C, and average relative humidity was slightly lower at 65%.

Locally, microclimatic monitoring was conducted at Point 1 using a stationary Davis Vantage Pro weather station. Throughout both monitoring periods, the station recorded consistently low wind speeds and no precipitation. During the wet period, the local station recorded mean air temperatures between 28 °C and 30 °C, and mean relative humidity between 59% and 75%. Compared with the official INMET baseline, these local readings showed higher mean air temperatures, indicating the distinct thermal impact of the park area. However, this discrepancy may be partially attributable to measurement timeframes: INMET data represent the entire daytime period, whereas concurrent local measurements were restricted to specific intervals within it. During the dry period, the local station recorded mean air temperatures of 29.7 °C to 31.3 °C and relative humidity of 62% to 74%. Overall, locally observed dry-season conditions closely mirrored the official INMET data, with only a slight increase in the local mean air temperature. Finally, differences in on-site relative humidity across seasons were negligible, though the average was higher in the dry season.

#### Wet season transect measurements and thermal stress

**Fig. 3:**
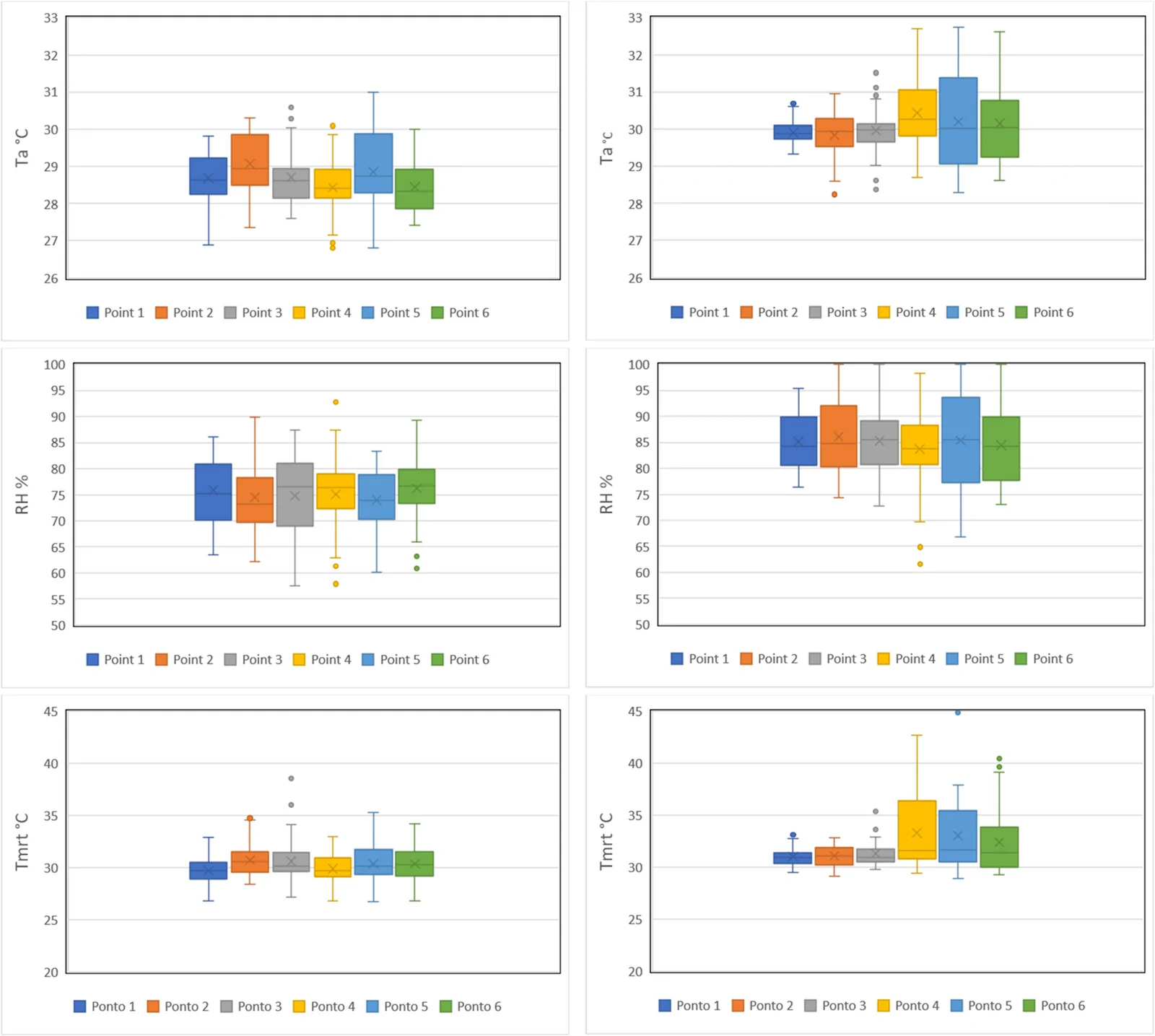
– Ambient temperature, relative humidity, and mean radiant temperature in the wet season (left column) and in the dry season (right column)

PLEMS measurements along the transect during the wet season (Fig. 3, left side) showed that the six sampling points had average air temperatures ranging from 28.4 °C to 29.1 °C. Point 2 recorded the highest average air temperature. In contrast, despite having the lowest sky view factor (SVF) and the densest vegetation, Point 5 exhibited the greatest temperature fluctuations and recorded the highest absolute maximum air temperatures. Consequently, its peak thermal stress measurements were driven by this extreme high variability rather than its mean temperature. Conversely, Points 4 (highest SVF) and 6 recorded the lowest average temperatures at 28.4 °C.

For relative humidity, Point 5 recorded the lowest average (74%), while Point 6 recorded the highest (76%). Point 5 also had the lowest maximum relative humidity (83%). In contrast, Point 4, which is more exposed to direct solar radiation and located near a pond, showed the highest maximum (93%). The lowest minimum relative humidity (57%) was observed at Points 3 and 4.

Air velocity remained low at all sampling points, consistently below 0.5 m/s.

Analysis of the mean radiant temperature (Tmrt) indicated that Point 1 had the lowest average (29.7 °C), while Point 2 had the highest (30.7 °C). These trends confirm that Point 2 was the warmest location along the route in terms of mean values, whereas Point 1 was the least warm. Point 5 exhibited the greatest fluctuation in Tmrt. The counterintuitive thermal behavior at Point 5, where high peak temperatures and extreme variability occurred despite dense shading, can be attributed to limited ventilation within the dense vegetation, which traps heat, combined with intermittent solar penetration through the canopy, causing sudden, sharp spikes in localized temperature readings.

More broadly during the wet season, the frequent presence of heavy cloud cover fundamentally alters the microclimatic dynamics between densely vegetated areas and open spaces. Under overcast conditions, the primary cooling benefit of a tree canopy, intercepting direct shortwave solar radiation, is largely neutralized as clouds diffuse the incoming sunlight. Consequently, spatial differences in ambient air temperature tend to decrease (as seen in the narrow 28.4–29.1 °C range), creating a more thermally uniform environment across the site. However, while air temperature gradients flatten, disparities in human thermal stress often widen. This divergence occurs because dense vegetation restricts localized airflow and traps moisture, creating stagnant, higher-humidity conditions that severely impede the evaporative cooling of the human body compared with more-ventilated open spaces. For example, in densely vegetated locations like Point 5, the canopy acts as a physical barrier that exacerbates this stagnant, humid microclimate. Without the convective cooling of wind or the ability to efficiently shed metabolic heat through sweat evaporation, these shaded areas can produce distinctly sultry conditions. As a result, vegetated points may paradoxically induce higher levels of thermal stress and discomfort compared to unshaded open sites during cloudy, wet periods.

**Fig. 4:**
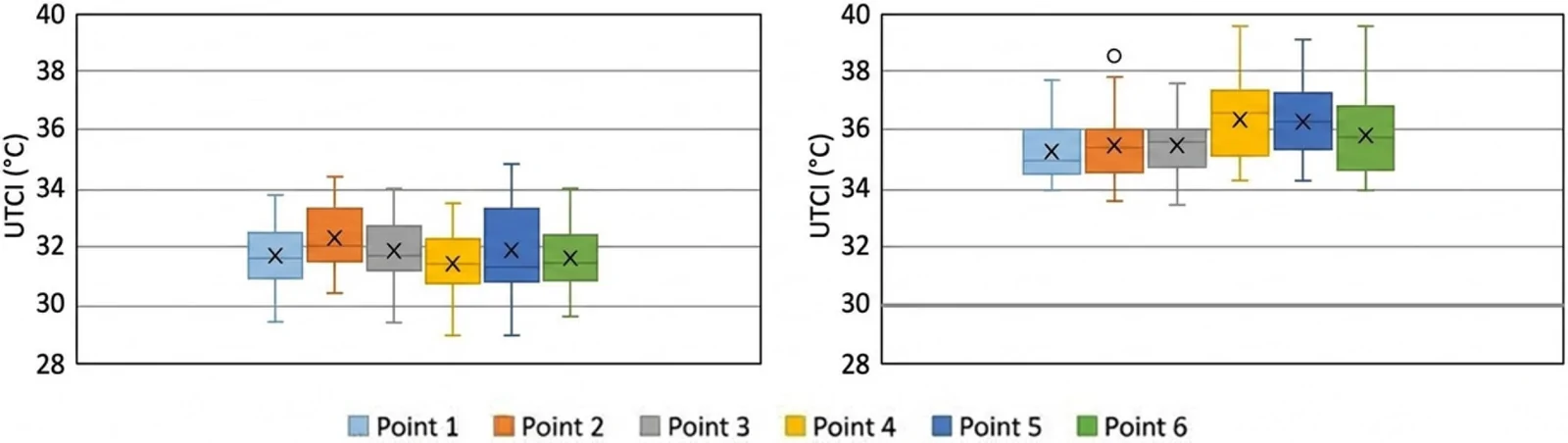
– UTCI outcomes for the wet season (left) and the dry season (right)

During the wet season (Fig. 4, left), the mean Universal Thermal Climate Index (UTCI) at the analyzed points was similar, ranging from 31.2 °C at Point 2 to 32.1 °C at Point 4. The lowest UTCI values (29.1 °C) were observed at Points 4 and 5, while the highest values were at Point 2 (33.9 °C) and Point 5 (34.3 °C). These thermal conditions correspond to the UTCI categories of moderate heat stress (29–32 °C) and strong heat stress (32.1–34 °C).

#### Dry season transect measurements and thermal stress

During the dry season (Fig. 3, right side), average air temperatures at the six points ranged from 29.8 °C at Point 2 to 30.4 °C at Point 4, representing a difference of only 0.6 °C in the mean. Based on trend values, Points 1 to 3 were the least warm, whereas Point 4 was the warmest overall. Both minimum and maximum temperatures increased compared to the wet season. Points 1 to 3 exhibited lower temperatures and fluctuations, while Points 4 to 6 exhibited higher temperatures and fluctuations. In contrast to the wet season, Point 4 recorded the highest maximum and average air temperatures during the dry season. Notably, this point also had the highest SVF, suggesting that increased solar radiation during the dry season may have contributed to this pattern, with a 30% increase in the mean global solar irradiance for the period.

Relative humidity varied minimally among the six points, with the lowest average at Point 4 (83.7%) and the highest at Point 2 (86.1%). Consistent with the wet season, air velocity remained low at all points, with averages below 0.5 m/s.

Average Tmrt increased substantially compared with the wet season. The highest average was recorded at Point 4 (33.3 °C). However, the maximum absolute value occurred as an isolated outlier at Point 5 (44.9 °C). This extreme Tmrt peak at the most densely vegetated point further suggests that momentary exposure to direct solar radiation through canopy gaps, paired with restricted local airflow, significantly drove up maximum readings during transit, even though the overall average remained lower. The lowest average was observed at Point 1. As with air temperature, Points 1 to 3 showed values clustered around their averages and medians, with low amplitude and lower overall values, particularly at Point 1. In contrast, Points 4 to 6 exhibited greater amplitude and higher values, with Point 4 identified as the warmest location along the route based on averages.

**Fig. 5:**
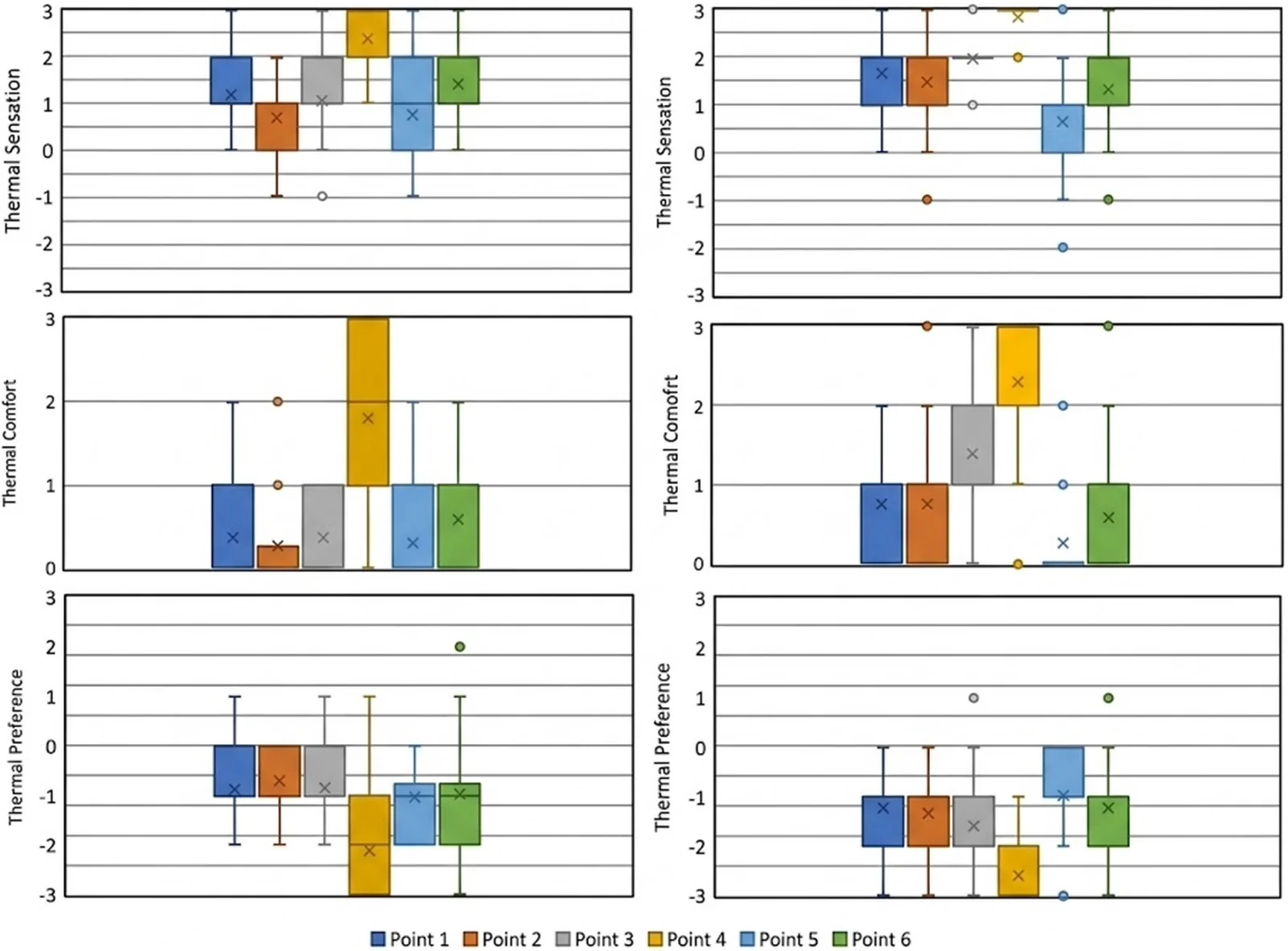
– Thermal sensation, thermal comfort, and thermal preference votes for the wet season (left) and the dry season (right)

In the dry season (Fig. 5, right), UTCI values were notably higher than those recorded during the wet season. The average UTCI values remained similar across the points, with the lowest, 34.6 °C, at Point 1 and the highest, 35.6 °C, at Points 4 and 5. The maximum value, 38.3 °C, was observed at Points 4 and 6, while the minimum value, 33.1 °C, occurred at Point 3. All points fell within the strong heat stress UTCI category (33.5 °C to 37 °C), with Points 4 and 6 nearing the very strong heat stress threshold at 38.3 °C.

### Subjective evaluation

During the wet period (Fig. 5, left), Point 2 was identified as the coolest location along the route, while Point 4 was perceived as the warmest. Point 2 exhibited one of the lowest sky view factors (SVF) at.174, combined with substantial shading from vegetation and permeable soil. In contrast, Point 4 had the highest SVF, resulting in the greatest solar irradiance. Solar radiation exposure at Point 4 was a key factor contributing to increased perceived thermal discomfort. The analysis of comfort perception aligns with the reported thermal sensation. Point 4 was consistently identified as the warmest and least comfortable location, whereas Point 2 was the coolest, with most responses indicating thermal comfort and some indicating slight or moderate discomfort. Reported thermal preferences further confirm Point 4 as the warmest, with a majority of respondents desiring a cooler or much cooler environment. In contrast, greater variability in opinions was observed for the other points. Points 1, 2, and 3 received similar evaluations, with an average preference for a slightly cooler environment.

However, this perceived order of warmth and thermal discomfort is not supported by the monitored data for each point during the wet season. Point 2 recorded the highest mean and median UTCI values, whereas Point 4 showed lower heat stress. The vegetated cover and overall characteristics of the forest environment at Point 2, compared with the unobstructed conditions at Point 4, may have contributed to a more favorable perception of the thermal environment.

Subjective perceptions during the dry season indicated greater thermal discomfort, attributable to higher in situ UTCI values measured with PLEMS than in the wet season. As shown in Fig. 5, both the mean and the first quartile at all measurement points fell within the ‘moderate heat stress’ category during the wet season. In contrast, dry season conditions shifted toward ‘strong heat stress,’ with maximum UTCI values at Points 4 and 6 reaching the ‘very strong heat stress’ category. At Point 4 in the dry season, participants predominantly reported a ‘very warm’ thermal sensation, with minimal outliers and first and third quartiles coinciding, indicating a consistent thermal response. These reports were accompanied by perceptions ranging from ‘very uncomfortable’ to ‘extremely uncomfortable’ and a thermal preference between ‘cooler’ and ‘much cooler.’ At Point 5, the prevailing thermal sensation was close to ‘slightly warm,’ with perceptions ranging from ‘comfortable’ to ‘slightly warm.’ Most participants reported feeling comfortable, with few outliers and identical first and third quartiles, suggesting a pronounced cooling effect and perceived thermal relief upon entering the heavily vegetated area. Thermal preference at Point 5 ranged from ‘neither warmer nor cooler’ to ‘slightly cooler’ within the quartiles.

To facilitate a direct comparison across the six measurement locations, all observations regarding physical site characteristics and subjective thermal perceptions have been integrated into a comprehensive summary table available in the Supplementary Materials. This table systematically synthesizes key environmental metrics, including site photographs, sky view factor (SVF), elevation gradients, pavement permeability, vegetation density, shading, and proximity to water bodies. Furthermore, it incorporates the primary findings from the wet and dry seasons for air temperature, relative humidity, mean radiant temperature, Universal Thermal Climate Index (UTCI) assessments, and corresponding subjective thermal responses. Consolidating this information provides a holistic overview of the climate walks and clarifies the distinct microclimatic profile of each designated point.

### Comparative analysis of thermal perception between consecutive points

**Fig. 6:**
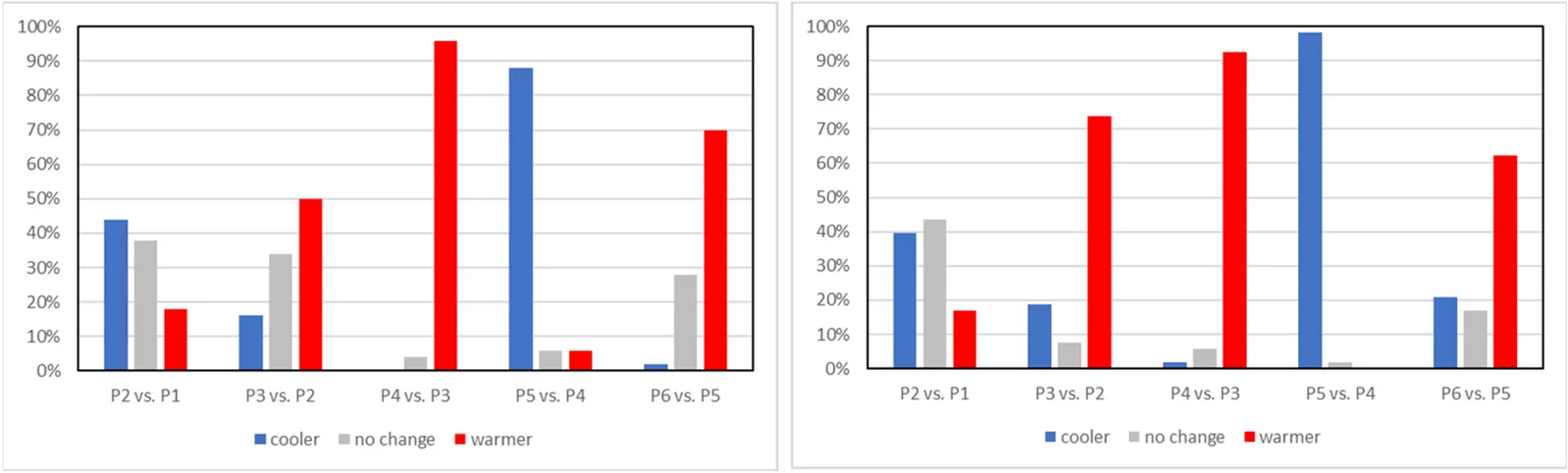
– Relative comparisons between points for the wet season (left) and the dry season (right)

Across both seasons, changes in sky view factor (SVF) due to increased vegetation and shading led to distinct variations in relative thermal perception between consecutive points (Fig. 6). Locations situated within denser tree canopies were consistently perceived as cooler, whereas more exposed sites were perceived as warmer. The most pronounced shifts in thermal perception occurred between Point 4 and Point 3 as participants exited the forest, and between Point 5 and Point 4 as they transitioned back into the forest from a highly exposed area.

Overall, these point-by-point comparisons closely aligned with the participants’ reported thermal sensations, perceptions, and preferences. Beyond direct microclimatic conditions, spatial factors such as vegetation density, ground cover, and solar radiation exposure strongly dictated thermal perception at each specific location. Points with lower SVF values due to increased tree canopy (Points 1, 2, and 5) experienced reduced solar radiation exposure and were perceived as less warm, even when local microclimatic measurements did not fully corroborate these subjective perceptions. Conversely, unshaded points with higher SVF values (Points 3, 4, and 6) were consistently perceived as warmer. Ultimately, these findings highlight the critical importance of employing a dynamic methodological approach. Capturing these immediate, point-to-point shifts in thermal perception enables a more accurate relative comparison of transient human comfort, revealing vital environmental nuances that static evaluations alone often fail to record.

### Correlation between microclimatic variables, subjective evaluation, and UTCI outcomes

As the qualitative (subjective responses) and quantitative (monitored data with PLEMS) variables did not follow a normal distribution according to the Shapiro-Wilk test at the 95% confidence level, Spearman’s rank correlation was used to assess associations between these variables. Statistical results are shown in Supplementary Materials – B.

Perceived temperature exhibited positive yet very weak, near-zero correlations with the microclimatic variables: air temperature (*r* = .122), the UTCI comfort index (*r* = .183), and relative air humidity (*r* = .166). While these coefficients indicate that increases in these variables mathematically correspond to a slight shift toward warmer thermal perceptions, their practical magnitude is highly limited.

Similarly, thermal comfort responses showed positive but weak correlations with air temperature (*r* = .120), relative air humidity (*r* = .125), and the UTCI index (*r* = .169), indicating a slight trend toward greater heat-related discomfort.

Thermal preference exhibited negative, low-magnitude correlations with relative air humidity (*r* = − .137) and the UTCI index (*r* = − .161), indicating a marginal shift toward a greater need for cooling under warmer conditions.

Overall, the correlations between subjective and microclimatic variables were consistently low, with coefficients approaching zero. Although these relationships are statistically significant (p-value < 0.05), this significance is primarily driven by the large sample size (𝑛 = 618) from a repeated-measures design rather than by a robust practical association; therefore, these linear metrics must be interpreted with caution. This inflated statistical power is an artifact of the repeated-measures design common to urban “climate walks,” where a sequence of individual votes is collected from the same participants at successive monitoring points in a stop-and-go fashion (Peng et al. 2022; Lau et al. 2026). Because such sequential exposures involve short-term thermal history, transient metabolic rates, and within-subject dependencies (Höppe 2002), these raw linear metrics must be interpreted with strict caution and should not be misconstrued as indicators of strong environmental determinism over subjective perception.

In urban thermal comfort literature, correlations ranging from *r* = .20 to 0.60 are more typical for raw data (e.g., Nikolopoulou and Lykoudis 2006; Krüger et al. 2011). For instance, Labdaoui et al. (2021) reported slightly higher correlations between thermal sensation votes and air temperature, and between thermal sensation votes and PET outcomes (*r* = .269 and *r* = .308, respectively). The particularly weak practical magnitude observed in this study may stem from the UTCI categorical scale’s lower resolution in hot and humid climates, which limits its predictive ability for acclimatized populations. Furthermore, these differences underscore that thermal evaluation is a highly subjective process influenced by a complex interplay of climatic, physical, physiological, and psychological factors (Nikolopoulou and Steemers 2003), which raw linear correlations cannot fully capture.

Conversely, the correlations among the subjective variables themselves were statistically significant and conceptually consistent. Negative correlations involving thermal preference and the variables of thermal sensation and comfort were expected, since preference represents a response in the opposite direction: greater discomfort from heat corresponds to a greater desire for cooler conditions.

### Confounding aspects and limitations of the study

To fully understand why the direct linear correlations with air temperature and UTCI remained weak in practice, two primary confounding factors warrant attention. The first concerns the potential underestimation of the impacts of solar radiation when relying solely on standard microclimatic metrics. Solar radiation emerged as a determining factor in subjective evaluations: points with a higher Sky View Factor (SVF), and consequently greater radiation exposure, were predominantly perceived as warmer, even when measured microclimatic variables did not fully capture these conditions. Conversely, points with lower SVF were perceived as less warm, effectively decoupling thermal perception from ambient air temperature.

The second factor involves the respondents’ transient thermal conditions and cumulative physical exertion. The route began with participants at rest and progressed through a 1,390 m walk. Despite intermittent stops, the protocol included only 5 min of stationary rest per point, which may have been insufficient for participants to achieve thermal equilibrium. The transition from inactivity to walking led to a progressive increase in metabolic rate and temporary heat storage, significantly confounding assessments of immediate thermal sensation and comfort. Notably, Point 6, representing the end of the route, featured the steepest incline, requiring participants to overcome approximately 10 m of elevation gain. This maximized metabolic heat generation immediately prior to their final evaluation.

Building upon these physiological dynamics, several methodological constraints regarding metabolic modeling and sample characteristics must be explicitly acknowledged. Primarily, the metabolic rate was set to 165 W/m², corresponding to a standard walking speed of 4 km/h per ISO 8896 (2004). Although our dynamic framework partially attributes shifts in thermal sensation to walking distance, terrain gradients, and body heat storage, it relies on a fixed metabolic rate, which represents a notable analytical limitation. Future studies should incorporate actual walking speeds, gradient data, and wearable physiological trackers (e.g., heart rate monitors) to estimate segment-specific metabolic rates. To overcome this constraint, subsequent work should leverage recent methodological advancements, such as transient UTCI and adjustment frameworks (ΔUTCI) that account for varying metabolic rates and higher activity levels beyond standard reference conditions (Lindner Cendrowska and Bröde 2021; Lindner Cendrowska et al. 2024). Integrating these approaches will enable more accurate dynamic thermal assessments by properly correcting for variable movement speeds and physical exertion during outdoor climate walks.

Furthermore, the study sample included diverse subgroups, such as overweight or obese participants, individuals reporting comorbidities, and a small number of subjects from different climatic backgrounds. Because body composition, health status, and prior climatic adaptation significantly influence individual thermal perception and physiological strain, these variables introduce potential confounding effects. Due to sample size limitations in these cohorts, comprehensive sensitivity analyses to isolate subgroup-specific patterns could not be performed.

Regarding environmental data, a distinct limitation involves the counterintuitive relative humidity (RH) patterns observed on site, including higher overall RH during the dry season. This discrepancy is primarily due to the diurnal timing of the measurement campaigns. It is important to emphasize that all reported schedules reflect local João Pessoa time rather than solar hours. Due to the city’s extreme eastern geographic position in Brazil, true solar noon occurs significantly earlier than 12:00 pm local time. Consequently, the wet season walks, which were heavily concentrated in the afternoon (mostly between 1:40 pm and 4:00 pm), closely aligned with the period of peak post-solar thermal accumulation. In contrast, dry season campaigns included morning sessions starting at 9:30 am. Because RH naturally peaks in the morning and drops during these afternoon thermal highs, the disproportionate frequency of afternoon wet season walks artificially depressed those averages relative to the dry season. Future studies should employ continuous, stationary monitoring to better isolate diurnal temporal variables from broader seasonal variations. Furthermore, to enable accurate pairwise comparisons across seasons or parts of the year, it is essential that future climate walk protocols strictly standardize walk time frames, ensuring that seasonal assessments are not confounded by predictable diurnal microclimatic shifts.

Finally, microclimatic data collection at each designated point was limited to brief five-minute observation windows. This yielded only about four records per location during the wet season and five during the dry season, meaning that exact temporal matching between individual questionnaire responses and their corresponding microclimatic observations cannot be guaranteed. Furthermore, because this field experiment was conducted within a single park under selected clear, low-wind daytime conditions, caution is warranted when generalizing the findings. Consequently, broader conclusions regarding the applicability of the Universal Thermal Climate Index (UTCI) in hot humid climates should be drawn with appropriate restraint.

## Conclusion

This study advances the understanding of thermal comfort dynamics in outdoor environments by deliberately departing from conventional single-point assessments. Rather than simply identifying the hottest or the coolest individual measurement point along the route, the primary contribution of this research lies in capturing the sequential thermal experience of pedestrians during a guided climate walk. By examining how the same participants experienced a continuous sequence of diverse microclimates and subjectively compared their current thermal state with that at the previous location, the study emphasizes the critical roles of environmental contrast and short-term thermal history in shaping transient thermal perception.

UTCI data consistently indicated a moderate level of heat-related thermal stress at all measurement points during both wet and dry periods. However, a comparison with participants’ subjective assessments revealed that the UTCI values indicated a higher heat stress level than perceived thermal sensation at two points during the wet period and underestimated it at three points during the dry period. The UTCI exhibits lower resolution in the heat-stress ranges, potentially limiting its predictive accuracy for thermal comfort in populations acclimatized to hot, humid climates. The greatest divergence was observed in the dry period, when ambient temperatures were higher.

Statistical analyses indicated that participants’ subjective evaluations weakly correlated with microclimatic variables, including air temperature and relative humidity, as well as with the derived UTCI thermal comfort index. Despite statistical significance, the low correlation coefficients suggest only a small magnitude of direct environmental determinism, reflecting the multifactorial nature of transient thermal perception. Two key dynamic factors explain these results. First, sudden environmental contrast significantly influenced perception; for instance, sharp changes in radiation exposure between heavily vegetated and exposed areas strongly shaped subjective evaluations, overriding absolute microclimatic variables. Second, participants’ accumulated metabolic heat throughout the continuous walk, transitioning from a resting stage to a sloped 1,390-meter light walk with reduced stationary time, led to progressive body heat storage. This transient thermal history compounded the immediate thermal sensation, an effect that intensified notably in the final segment due to a steep incline, resulting in greater perceived warmth and discomfort regardless of the local microclimate.

Consequently, future dynamic thermal comfort studies must carefully integrate these temporal variables. We therefore highlight the importance of incorporating adequate resting stops during climate walks when conducting interpoint comparisons. These pauses are essential to allow participants to approach thermal equilibrium and dissipate the metabolic heat accumulated from continuous physical exertion. Furthermore, it is crucial to account for uneven terrain with steep inclines and declines. Such spatial transitions substantially alter metabolic rates and ultimately dictate the sequential thermal experience of research participants. Ultimately, by addressing these methodological factors, this study contributes to the establishment of refined protocols for climate walks, improving the assessment of the thermal benefits and restorative functions of urban green areas. Participants were asked to sign a consent form, declaring that they understood the aims of the study and agreed to the terms of the questionnaire survey. Solange Leder – conceptualization, methodology, supervision, writing. Eduardo Krüger (corresponding author) – analysis, writing, final revision.

## Supplementary Information

Below is the link to the electronic supplementary material.

Supplementary Material 1

## Data Availability

The datasets generated during the current study are available from the corresponding author on reasonable request.
