# Supplementary material for: Dynamic evaluation of outdoor thermal comfort in an urban park situated in a tropical region
Source: Int J Biometeorol. 2026 Oct 30;70(11):290. doi: 10.1007/s00484-026-03359-2 (PMC13722759; doi:10.1007/s00484-026-03359-2)
Supplement: Supplementary file 1 [file 484_2026_3359_MOESM1_ESM.docx]

**Dynamic evaluation of outdoor thermal comfort in an urban park situated in a tropical region – SUPPLEMENTARY MATERIALS**

Appendix A – Climatic conditions

**Table A.** Overall climatic conditions recorded during the wet season, including total daily precipitation (Precip, mm), total daily global solar irradiance (Ig, kJ/m²), minimum and maximum ambient temperatures (Tmin, Tmax, °C), and average relative humidity (%) during daytime monitoring hours.

| **Date** | **Precip (mm)**  **daily total** | **Ig (kJ/m²)**  **daily total** | **T min (°C)**  **daytime** | **T max (°C)**  **daytime** | **RH (%)**  **daytime** |
| --- | --- | --- | --- | --- | --- |
| 9-Jul | 2 | 19534 | 23.3 | 29.5 | 73 |
| 11-Jul | 0 | 17439 | 21.5 | 29.4 | 70 |
| 13-Jul | 4 | 20040 | 22.7 | 29.9 | 71 |
| 15-Jul | 0 | 13717 | 24.1 | 29.3 | 71 |
| 18-Jul | 6 | 14506 | 23.0 | 29 | 75 |
| 21-Jul | 0 | 19807 | 23.6 | 29.3 | 62 |
| 23-Jul | 1 | 19702 | 22.2 | 29.0 | 64 |
| 29-Jul | 0 | 20839 | 20.9 | 30.6 | 65 |
| 30-Jul | 3 | 16542 | 23.5 | 29.2 | 76 |
| 6-Aug | 6 | 19109 | 24.6 | 30.1 | 66 |
| 10-Aug | 0 | 19062 | 21.8 | 29.0 | 68 |

**Table B.** Overall climatic conditions recorded during the dry season, including total daily precipitation (Precip, mm), total daily global solar irradiance (Ig, kJ/m²), minimum and maximum ambient temperatures (Tmin, Tmax, °C), and average relative humidity (%) during daytime monitoring hours.

| **Date** | **Precip (mm)**  **daily total** | **Ig (kJ/m²)**  **daily total** | **T min (°C)**  **daytime** | **T max (°C)**  **daytime** | **RH (%)**  **daytime** |
| --- | --- | --- | --- | --- | --- |
| 27-Dec | 0 | 19879 | 26.2 | 31.4 | 67 |
| 28-Dec | 0 | 23652 | 26.6 | 31.6 | 61 |
| 4-Jan | 0 | 24105 | 26.2 | 31.1 | 67 |
| 6-Jan | 0 | 21522 | 26.9 | 31.4 | 65 |
| 7-Jan | 0 | 26449 | 24.9 | 31.3 | 62 |
| 9-Jan | 0 | 25983 | 26.7 | 31.3 | 65 |
| 13-Jan | 0 | 24278 | 26.2 | 31.1 | 69 |
| 14-Jan | 0 | 20815 | 27.0 | 30.9 | 66 |
| 20-Jan | 0 | 26084 | 25.4 | 31.7 | 65 |

Appendix B – Statistical tests

Table C – Normality test for microclimatic data

| **Shapiro-Wilk Test**  **p > .05** | **Ta (°C)** | **Tg (°C)** | **RH (%)** | **v (m/s)** | **Tmrt (°C)** |
| --- | --- | --- | --- | --- | --- |
| W-stat | .966004 | .962635 | .979445 | .237891 | .939178482 |
| p-value | < .01 | < .01 | < .01 | < .01 | < .01 |
| alpha | .05 | .05 | .05 | .05 | .05 |
| normal | No | No | No | No | No |

Table D – Normality test for subjective responses

| **Shapiro-Wilk Test**  **p > .05** | **Thermal Sensation** | **Thermal Comfort** | **Thermal Preference** | **Relative thermal sensation**  **regarding the previous Point** |
| --- | --- | --- | --- | --- |
| W-stat | .90869 | .730583 | .851262 | .80646 |
| p-value | < .01 | < .01 | < .01 | < .01 |
| alpha | .05 | .05 | .05 | .05 |
| normal | No | No | No | No |

Table E – Spearman correlations between subjective responses, microclimatic variables, and UTCI outcomes

| **Microclimatic variable / comfort index** | **Thermal Sensation** | **Thermal Comfort** | **Thermal Preference** |
| --- | --- | --- | --- |
| **Ta (°C)** | .122* | .120* | -.095* |
|  | p = .002 | p = .003 | p = .018 |
| **Tg (°C)** | -.044 | .054 | -.026 |
|  | p = .277 | p = .179 | p = .524 |
| **RH (%)** | .166** | .125* | -.137** |
|  | p = .000 | p = .002 | p = .001 |
| **v (m/s)** | .008 | .018 | .033 |
|  | p = .839 | p = .674* | p = .407* |
| **Tmrt (°C)** | .029 | .041 | -.007 |
|  | p = .476 | p = .309 | p = .863 |
| **UTCI (°C)** | .183** | .169** | -.161** |
|  | p = .000 | p = .000 | p = .000 |

*statistically significant results at p < .05 (*) and at p < .01 (**)*

Table E – Spearman correlations between subjective responses

| **Subjective variable** | **Thermal Sensation** | **Thermal Comfort** |
| --- | --- | --- |
| **Thermal Comfort** | .730** |  |
|  | p = .000 |  |
| **Thermal Preference** | -.537** | -.667** |
|  | p = .000 | p = .000 |

*statistically significant results at p < .05 (*) and at p < .01 (**)*

Table F – Comprehensive Summary of Measurement Points, Environmental Characteristics, and Thermal Results

| **Parameter / Feature** | **Point 1 (P1)** | **Point 2 (P2)** | **Point 3 (P3)** | **Point 4 (P4)** | **Point 5 (P5)** | **Point 6 (P6)** |
| --- | --- | --- | --- | --- | --- | --- |
| **Location & Context** | Beginning of park trail | Trail segment in forested area | Near park gathering area, snack bars, train station | Near park pond, open area | Wetland area in park's lowest terrain | Final point near recreation area (uphill incline) |
| **Distance & Elevation Change** | (Start) | 300 m / +1 m | 280 m / -13 m | 210 m / -8 m | 410 m / +2 m | 190 m / +10 m |
| **Sky View Factor (SVF)** | 0.403 | 0.174 | 0.634 | 0.828 | 0.092 | 0.282 |
| **Pavement Type & Permeability** | Cobblestones (Impermeable) | Compacted sandy soil (Permeable) | Interlocking concrete paving (Impermeable) | Interlocking concrete paving (Impermeable) | Compacted soil, stones, grass, wooden walkways (Mixed/Permeable) | Granite cobblestones (Impermeable) |
| **Vegetation Density & Characteristics** | Robust; shrubs & trees | High diversity; shrubs & trees lining trail | Sparse; shrubs & trees | Very sparse; grassy spaces, few trees | Dense; medium- and large-sized trees (wetland) | Tall but not dense tree vegetation |
| **Shading & Solar Exposure** | Extensive shade | Extensive shade | Alternating sunlit and shaded areas | Highest exposure to solar radiation | Most shaded point along route | Shaded and sunlit areas |
| **Proximity to Water** | None | None | Adjacent to park facilities | Adjacent to a pond | Located in wetland crossing small watercourses | None |
| ***Wet Season Results*** | | | | | | |
| **Ta (°C)** | Avg ~29.4 (28.0–30.2) | Avg ~29.8 (28.2–30.6) | Avg ~29.3 (28.3–30.4) | Avg ~29.2 (27.7–30.4) | Avg ~29.2 (27.9–30.8) | Avg ~29.1 (28.1–29.9) |
| **RH (%)** | Avg ~78% (67–88%) | Avg ~77% (66–88%) | Avg ~77% (62–88%) | Avg ~78% (62–93%) | Avg ~74% (65–83%) | Avg ~76% (65–91%) |
| **Tmrt (°C)** | Avg ~29.7 (29.2–31.4) | Avg ~30.7 (29.6–32.3) | Avg ~30.7 (29.7–32.7) | Avg ~30.9 (29.7–34.3) | Avg ~31.2 (30.1–33.0) | Avg ~31.1 (30.0–32.2) |
| **UTCI (°C)** | Avg ~31.6 (29.4–33.7) | Avg ~32.5 (30.4–34.4) | Avg ~31.8 (29.4–33.9) | Avg ~31.4 (29.0–33.5) | Avg ~32.0 (29.0–34.8) | Avg ~31.5 (29.6–34.0) |
| **Subjective Responses** | Sensation: Warm (+1 to +2)  Comfort: Comfortable to slight discomfort  Preference: Slightly cooler | Sensation: Neutral to warm (perceived as coolest)  Comfort: Comfortable  Preference: Slightly cooler | Sensation: Warm (+1 to +2)  Comfort: Comfortable to slight discomfort  Preference: Slightly cooler | Sensation: Very warm (+2 to +3)  Comfort: Very/Extremely uncomfortable  Preference: Much cooler | Sensation: Warm (+1 to +2)  Comfort: Comfortable to slight discomfort  Preference: Slightly cooler | Sensation: Warm (+1 to +2)  Comfort: Comfortable to slight discomfort  Preference: Slightly cooler |
| ***Dry Season Results*** | | | | | | |
| **Ta (°C)** | Avg ~29.5 (28.5–30.0) | Avg ~29.8 (28.2–30.4) | Avg ~29.5 (28.3–30.6) | Avg ~30.4 (28.5–31.4) | Avg ~29.8 (28.3–31.4) | Avg ~29.6 (28.5–30.1) |
| **RH (%)** | Avg ~86% (79–96%) | Avg ~87% (77–100%) | Avg ~86% (75–100%) | Avg ~84% (70–100%) | Avg ~86% (70–100%) | Avg ~85% (75–96%) |
| **Tmrt (°C)** | Avg ~31.0 (30.4–32.0) | Avg ~31.0 (30.2–32.0) | Avg ~31.0 (30.2–32.3) | Avg ~33.3 (30.9–38.3) | Avg ~31.7 (30.4–44.9) | Avg ~31.4 (30.2–33.4) |
| **UTCI (°C)** | Avg ~35.3 (34.0–37.7) | Avg ~35.5 (33.6–38.6) | Avg ~35.5 (33.4–37.6) | Avg ~35.6 (34.3–39.6) | Avg ~35.6 (33.9–39.1) | Avg ~35.6 (33.9–39.6) |
| **Subjective Responses** | Sensation: Warm (+1 to +2)  Comfort: Comfortable to slight discomfort  Preference: Cooler | Sensation: Warm (+1 to +2)  Comfort: Comfortable  Preference: Cooler | Sensation: Warm to hot (+1 to +3)  Comfort: Moderate discomfort  Preference: Cooler | Sensation:  Very warm (+2 to +3)  Comfort: Very/Extremely uncomfortable  Preference: Much cooler | Sensation: Slightly warm (perceived relief)  Comfort: Comfortable  Preference: Slightly cooler | Sensation: Warm (+1 to +2)  Comfort: Comfortable to slight discomfort  Preference: Cooler |
